# Line-Based Registration of Panoramic Images and LiDAR Point Clouds for Mobile Mapping

**DOI:** 10.3390/s17010070

**Published:** 2016-12-31

**Authors:** Tingting Cui, Shunping Ji, Jie Shan, Jianya Gong, Kejian Liu

**Affiliations:** 1 State Key Laboratory of Information Engineering in Surveying Mapping and Remote Sensing, Wuhan University, Wuhan 430079, China; cuitingting@whu.edu.cn (T.C.); gongjy@whu.edu.cn (J.G.); 2 Research Center of Remote Sensing in Public Security, People’s Public Security University of China, Beijing 100038, China; liukejian@ppsuc.edu.cn; 3 School of Remote Sensing and Information Engineering, Wuhan University, Wuhan 430079, China; 4 RSISE, Australian National University, Canberra, ACT 2600, Australia; 5 Collaborative Innovation Center of Geospatial Technology, Wuhan University, Wuhan 430079, China; 6 Lyles School of Civil Engineering, Purdue University, West Lafayette, IN 47907, USA

**Keywords:** mobile mapping system, LiDAR point cloud, 2D-3D registration, panoramic sensor model

## Abstract

For multi-sensor integrated systems, such as the mobile mapping system (MMS), data fusion at sensor-level, i.e., the 2D-3D registration between an optical camera and LiDAR, is a prerequisite for higher level fusion and further applications. This paper proposes a line-based registration method for panoramic images and a LiDAR point cloud collected by a MMS. We first introduce the system configuration and specification, including the coordinate systems of the MMS, the 3D LiDAR scanners, and the two panoramic camera models. We then establish the line-based transformation model for the panoramic camera. Finally, the proposed registration method is evaluated for two types of camera models by visual inspection and quantitative comparison. The results demonstrate that the line-based registration method can significantly improve the alignment of the panoramic image and the LiDAR datasets under either the ideal spherical or the rigorous panoramic camera model, with the latter being more reliable.

## 1. Introduction

Motivated by applications such as vehicle navigation [1], urban planning [2], and autonomous driving [3], the need for 3D detailed photorealistic models in urban areas has increased dramatically in recent years. A mobile mapping system (MMS), which collects 3D and/or 2D photographic data while vehicles move at a regular speed, have been widely used as the efficient data acquisition technology at street-level [4]. Short range laser scanners (e.g., 100–200 m) and electro-optical cameras are two primary sensors on a MMS, each of which has its own characteristics. Laser scanners can acquire 3D information directly, but offer relatively low resolution and short range of use, whereas a camera captures 2D images with high-resolution textures but the depth information is missing. Thus, these complementary characteristics between ranging and imaging sensors have been broadly studied through the so-called camera/LiDAR fusion [5,6,7,8].

A prerequisite of data fusion is to transform different datasets to a common coordinate system, which is often called 2D-to-3D registration. Although the camera and LiDAR sensors are usually calibrated [9,10,11,12] in advance, considerable misalignment may still exist between the two datasets. Possible reasons for such misalignment are as follows: (1) System calibration errors. The relative orientation and position offsets between all the sensors, GPS, Inertial Measurement Unit (IMU), LiDAR and camera on a MMS, may lack precise measurements by the manufacturer. Further, the offsets and relative orientations determined prior to data collection may change due to mechanical vibrations [13]; (2) Different acquisition time. The image is collected at a certain exposure time while the corresponding point cloud data are actually collected via continuous scanning during a period of time, indicating that the two sensors are not strictly synchronized; (3) Unreliable GPS signals make this even worse. At street level, the GPS signals often suffer from ambiguity and loss due to multipath and canyon effects. Even when IMU, Distance Measuring Indicator (DMI) or Different GPS (DGPS) are onboard, noticeable co-registration errors may still exist. All of the above unavoidable factors call for data-driven registration methods in the subsequent data processing procedure.

There have been a number of related studies pertaining to 2D-3D registration, and the reader is referred to the review in [14] for a comprehensive discussion of them. The registration framework for LiDAR and optical images [15], as outlined in the concept of image registration [16], includes four components: registration primitives, similarity measure, registration transformation, and matching strategy.

As to the first component, we use straight-line as the registration primitives in this paper for two reasons. First, linear features have the potential to be reliably, accurately, and automatically extracted from both 2D images and 3D point clouds. While the former has been well studied [17,18,19], the latter has received relatively less attention. Early studies on 3D line segments usually extracted plane intersection lines [20,21], and more recent work also considered depth discontinuity lines [22,23]. Applying a 2D line detection algorithm on a set of shaded images rendered from a point cloud with different views, reference [24] provided reasonable results of 3D line segments from an unorganized point cloud. And specifically, some studies also focused on linear objects such as poles [25,26] and curbs [27,28]. Second, the methods using linear features exceed those using point features on registration accuracy, which is demonstrated in the technical report of the “Registration Quality—Towards Integration of Laser Scanning and their Performance” project, sponsored by European Spatial Data Research (EuroSDR), 2008–2011 [29].

The principles and concepts of line-based registration originated from the highly researched topic of line photogrammetry in the late 1980s and early 1990s whereby linear features, especially straight lines, were regarded as basic an entity as traditional point features. Reference [30] described the concepts, mathematical formulations, and algorithms related to line photogrammetry, which is based on the straight lines extracted from digital images. In [31] a collinearity model to establish the relationship between lower level features, such as edge pixels, instead of fitting lines in the image space and the control lines in the object space was proposed. Reference [32] proposed the concept of “generalized points,” a series of connected points representing a linear feature. According to this concept, the traditional collinearity model can accommodate more complicated features, such as circles and rectangles, rather than only straight lines. The authors in [20,21] utilized linear features to determine camera orientation and position relative to a 3D model. Vanishing points extracted from 2D images and 3D principle directions derived from a 3D model were also used to estimate camera orientation. In addition, there were also many studies using statistical similarity measures for the registration of the LiDAR points and images. Reference [33] developed a registration strategy based on global mutual information and exploited the statistical dependency between the intensity and measured LiDAR elevation, while [34] investigated the effectiveness of both local and global mutual information.

All of the above studies were based on the popular frame cameras. However, a panoramic camera rather than a frame camera is used in our MMS, which means a new panoramic sensor model must be taken into account. The greatest advantage of a panoramic camera is its 360° view angle, essentially making it a standard component in recently produced MMS. The online street-view maps provided by Google, Microsoft, and Baidu and Tencent were mostly generated from geo-registered panoramic imagery. Unfortunately, none of the limited studies on LiDAR and image registration involving panoramic cameras considered a rigorous panoramic camera model. References [8,35] proposed an automatic registration of mobile LiDAR and spherical panoramas; however, only part of the panoramic image in the limited viewport was used. Moreover, the registration was based on a conventional frame camera model.

Besides registration, many applications based on panoramic images have been reported in recent years. In [36] a structure-from-motion (SfM) pipeline for 3D modeling using Google Street View imagery with an ideal spherical camera model was presented, while [37] presented a piecewise planar model to reconstruct 3D scenes from panoramic image sequences. A quadrangular prism panoramic camera model was used for improved image matching. Reference [38] explained both the ideal spherical camera model and the rigorous panoramic sensor model for a multi-camera rig and their corresponding epipolar geometry. In [39] the two models were further compared and their effects on the localization quality in object space and the quality of space resection. In this paper, we utilize the rigorous panoramic sensor model for image and LiDAR registration. Since there were many studies based on the ideal spherical camera model, we also attempt to demonstrate its limitations in this paper by comparing it with the rigorous model.

This paper proposes a rigorous line-based registration method for precise alignment of LiDAR point clouds and panoramic images. The remainder of this paper is structured as follows: Section 2 introduces a MMS and its sensor frames for the LiDAR and the panoramic camera. Section 3 presents registration models based on straight-line features for panoramic cameras. Section 4 addresses 3D line extraction from LiDAR point clouds. Section 5 introduces the datasets and analyzes the registration results based on our model. Finally, the conclusions and future work recommendations are presented in Section 6.

## 2. The Mobile Mapping System and Sensor Configuration

**Figure 1:**
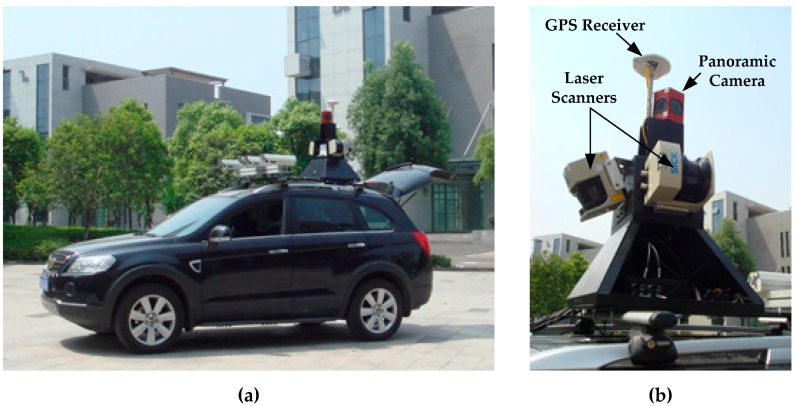
The MMS used in this study: (**a**) the vehicle; (**b**) the panoramic camera, laser scanners, and GPS receiver.

The MMS used in this paper was jointly developed by Wuhan University and Leador Spatial Information Technology Corporation, Ltd. (Wuhan, China), configured with a Ladybug 3 camera [40], three low-cost SICK laser scanners [41] (one for ground and two for facades) and a GPS/IMU. The system and its sensor configuration are shown in Figure 1.

This section first introduces several key coordinate systems of the MMS and their geometric relationships, followed by a description of the geo-referenced LiDAR and the rigorous camera model for a multi-camera rig.

### 2.1. Coordinate Systems

**Figure 2:**
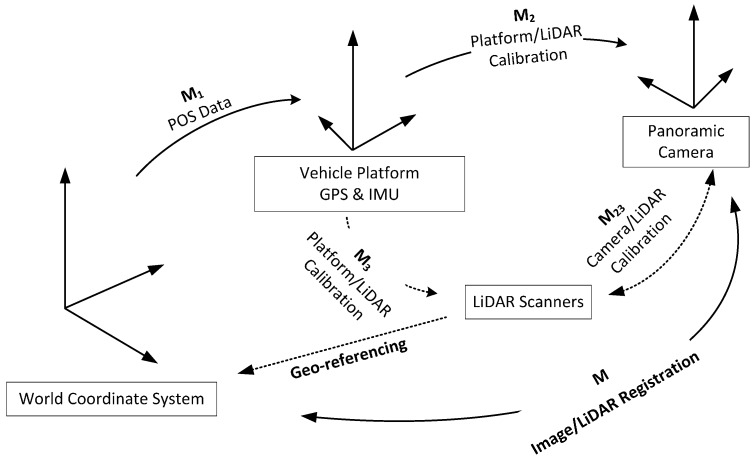
Coordinate systems of the Mobile Mapping System.

As shown in Figure 2, there are three coordinate systems in our MMS: (1) a world coordinate system; (2) a vehicle platform coordinate system; and (3) a camera-centered coordinate system. The world coordinate system is the reference for data management and organization. In the proposed method, the GPS/IMU records the translation and orientation from the world coordinate system to the vehicle platform coordinate system denoted as ***M***_1_(***R***_1_, ***T***_1_). The LiDAR points are geo-referenced to the world coordinate system (the left-bottom dotted line) according to ***M***_1_ and the calibration parameters ***M***_3_ between the LiDAR sensor and the platform. ***M***_2_(***R***_2_, ***T***_2_) is the transformation from the camera to the vehicle platform, which is also achieved through prior calibration.

The goal of registration is to determine the transformation ***M*** from the LiDAR points to the panoramic image. Other than a static calibration which concerns only ***M***_23_, the time series of localization information ***M***_1_ is considered. For simplification, the possible errors of ***M***_3_ are ignored and the transformation is constructed directly between the georeferenced LiDAR and camera (the bottom solid line). It is assumed that there exists ***ΔM***(Δ***R***, Δ***T***) to meet:

(1) $$M=\left[\begin{array}{cc} ∆R & ∆T\\ 0 & 1 \end{array}\right]\left[\begin{array}{cc} R_{2} & T_{2}\\ 0 & 1 \end{array}\right]\left[\begin{array}{cc} R_{1} & T_{1}\\ 0 & 1 \end{array}\right]$$

***ΔM*** compensates for several aspects including ***M***_3_ (as is discussed in Section 1). The line features are extracted from both the images and the LiDAR points, which are then utilized to determine the optimal ***ΔM*** for an accurate registration. The solution procedure is based on the standard least squares technique imbedded with RANSAC for removal of possible gross errors in ***M***_1_.

### 2.2. Geo-Refereneced LiDAR

The LiDAR points are geo-referenced to the world coordinate system by the interpolated rotation values recorded by INS at the corresponding position from the GPS/IMU integrated navigation data ***M***_1_ and the calibration parameters ***M***_3_ between the LiDAR and the IMU [42]. In the proposed system, three low-cost SICK laser scanners (all linear-array lasers) are equipped to acquire a 3D point cloud of the object’s facade. The angular resolution (0.25°–1.0°) and scan frequency (25–75 Hz) are fixed during data acquisition. The density of LiDAR points is uneven, i.e., the closer they are to the measured surface, the higher the density of the points. For instance, the points on the ground are much denser than those on the top facade. In addition, the point density in the horizontal direction is dependent on the velocity of the MMS vehicle.

### 2.3. Multi-Camera Rig Models

**Figure 3:**
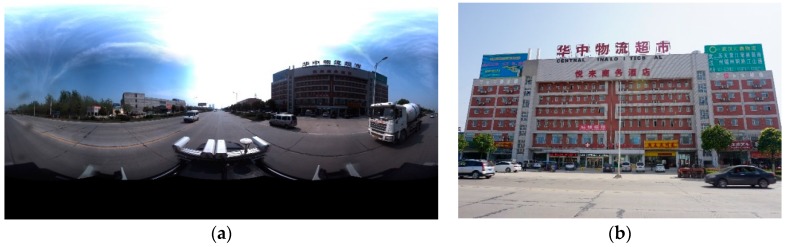
Comparison between (**a**) a panoramic image and (**b**) a frame image. (The meaning of the Chinese characters on the building is Supermarket for Logistics in Central China).

The panoramic image in Figure 3 covers a 360° view of a surrounding scene, captured by the Ladybug 3 system composed of six fisheye cameras. The straight lines in reality are no longer straight on panoramic images compared to a common frame Figure 3b.

Generally, the panoramic imaging process can be approximated by an ideal spherical camera model. However, since the entire image is technically stitched from six individual images through blending, stitching errors cannot be avoided. This section first introduces the traditional ideal spherical camera model and then extends it to the rigorous multi-camera rig panoramic model. The spherical camera model is referred to as the ideal one and the panoramic camera model as the rigorous one.

#### 2.3.1. Spherical Camera Model

**Figure 4:**
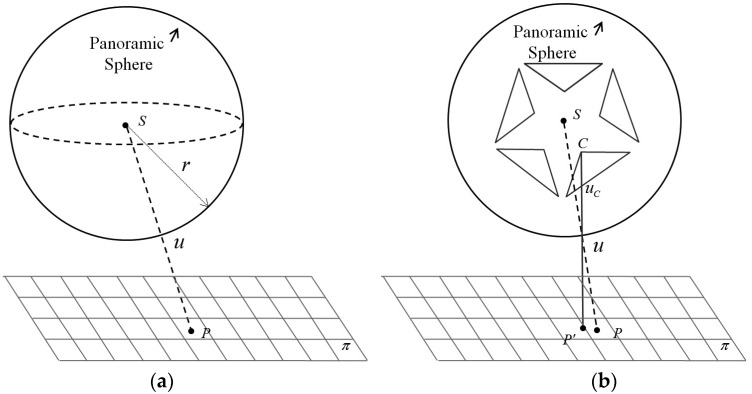
Differences between the spherical and panoramic camera models. (**a**) The dashed line shows the ray through 3D point ***P***, panoramic image point ***u***, and sphere center ***S***; (**b**) the solid line shows the ray through 3D point ***P***′, mono-camera image point ***u_c_***, panoramic image point u, and the mono-camera projection center ***C***.

Under this model, the imaging surface is regarded as a sphere, whose center is the projection center. Figure 4a presents a schematic diagram of the spherical projection, where the sphere center ***S***, the 3D points ***P*** in plane *π*, and the panoramic image point ***u*** are collinear [39]. The pixels in a panoramic image are typically expressed in polar coordinates. Assuming that the width and height of the panoramic image is *W* and *H* respectively, the horizontal 360° view is mapped to [0, *W*] and the vertical 180° view is mapped to [0, *H*]. Thus, each pixel (*u*, *v*) can be transformed to polar coordinate (θ,φ) by Equation (2):

(2) $$\{\begin{array}{c} \theta =\left(2u-W\right)\cdot \frac{\pi }{W}\\ \varphi =\left(1-\frac{2v}{H}\right)\cdot \frac{\pi }{2} \end{array}$$

θ is the horizontal angle between −*π* and *π*, and φ is the vertical angle between −*π/2* and *π/2*. Let *r* be the radius of the projection sphere, Equation (3) is used to calculate a set of Cartesian coordinates. In most cases, *r* = 20.0 m for the best stitching accuracy [43].

(3) $$\{\begin{array}{c} x=r\cdot \cos\;\varphi \cdot \sin\;\theta \\ y=r\cdot \cos\;\varphi \cdot \cos\;\theta \\ z=r\cdot \sin\;\varphi \end{array}$$

The sphere center ***S***, 3D point ***P***, and edge pixel ***u*** are collinear. The relationship between ***X*** and ***P*** is established by perspective transformation in Equation (4):

(4) $$X=\;\lambda^{-1}R^{T}\left(P-T\right)$$

where ***P*** is the coordinate of the object point, and ***X***(*x*, *y*, *z*) is the Cartesian coordinate of image point ***u***; ***R*** and ***T*** are respectively the rotation matrix and translation vector between the object space and the panoramic camera space; and λ is the scale factor.

Unlike the traditional camera model, the z coordinate of the image point is not equal to *−f*, where *f* is the focal length in the traditional camera model. In the widely used spherical camera model, the image point *u* is under the spherical geometry constraint as Equation (5):

(5) $$x^{2}+y^{2}+z^{2}=r^{2}$$

As a result, Equation (4) actually has two degrees of freedom, i.e., two independent equations.

#### 2.3.2. Panoramic Camera Model

A multi-camera rig consists of several separate and fixed fish-eye lenses. Independent images are captured by each lens and then stitched to form the entire panoramic image. As shown in Figure 4b, each lens has its own projection center ***C***, but it cannot be precisely located on sphere center ***S*** due to the manufacturing constraints. The mono-lens center ***C***, instead of sphere center ***S***, panoramic image point ***u***, and 3D point ***P′*** in object space are collinear.

**Figure 5:**
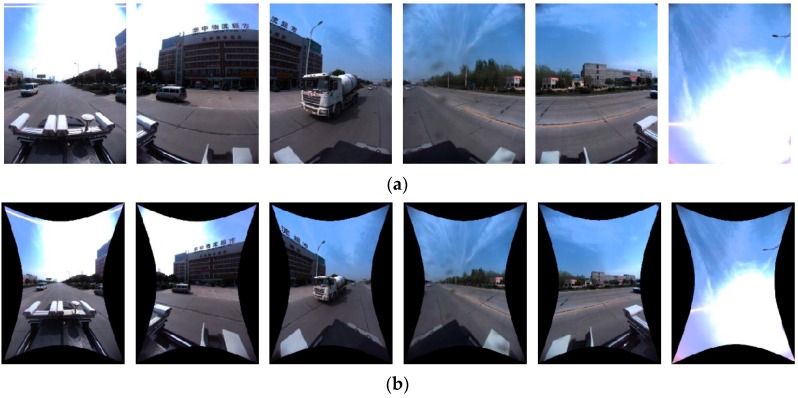
Images of Camera 0–5: (**a**) 6 fish-eye images; (**b**) 6 rectified images. (The meaning of the Chinese characters on the building is Supermarket for Logistics in Central China).

The panoramic camera used in this paper is composed of six separate fish-eye lenses. Figure 5a shows an example of the six raw fisheye images, and Figure 5b shows the corresponding undistorted images rectified from the raw ones. Rectification from a fisheye to an ideal plane image only depends on the known camera calibration parameters ***Kr***, including the projection model and the radial and tangential distortion. The index r means that every fisheye camera has its own calibration parameters. Since the straight lines, such as the boundaries of buildings, are distorted in the raw fisheye image, rectified images are used for line extraction.

**Figure 6:**
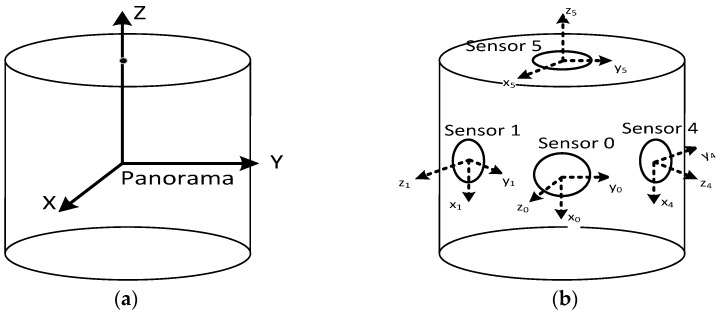
Global and local coordinate systems of multi-camera rig under a cylindrical projection. (**a**) the global panoramic camera coordinate system and (**b**) six local coordinate systems of the rectified cameras.

As shown in Figure 6, the global camera coordinate system is defined for the whole multi-camera rig, and six local coordinate systems are defined for each lens separately. The global coordinate system (see Figure 6a) of the panoramic camera is defined by three main directions: the X-axis typically is along the driving direction; the Z-axis is the zenith direction; and the Y-axis is orthogonal to both the Y-axis and the Z-axis. Each of the six lenses has its own local coordinate system (see Figure 6b): (1) The origin is the optical centre of the lens; (2) the Z-axis is the optical axis and points towards the scene; and (3) the X-axis and the Y-axis are parallel to the corresponding image coordinate. For each lens, there are three interior orientation elements (focal length *f* and image centre (*x***_0_**, *y***_0_**)) and six exterior orientation parameters (*T_x_*, *T_y_*, *T_z_*, *R_x_*, *R_y_*, *R_z_*) relative to the global coordinate system (the offsets between ***C*** and ***S*** in Figure 4b under spherical view). Both of them were acquired in advance by careful calibration by the manufacturer.

Although each lens may have its own camera model, the advantages of panoramic imaging may be overlooked. To address this issue, all six images are projected into the global spherical imaging surface to obtain uniform coordinates. First, the coordinates of the rectified image of each separate lens are transformed to the global coordinate system. Each pixel ***p*** (*x*, *y*) in the rectified images forms a 3D ray in the global coordinate system by Equation (6):

(6) $$X^{\prime }=mR_{r}X_{r}+T_{r}.$$

where ***X_r_*** (*x* − *x*_0_, *y* − *y*_0_, *f*) is the mono-camera coordinates of pixel ***p*** and the translation vector ***T_r_*** (*T_x_*, *T_y_*, *T_z_*) and the local rotation matrix ***R_r_*** are known, the latter can be calculated by the following equation:

(7) $$R_{r}=\{\begin{array}{ccc} \cos R_{z}\cos R_{y} & \cos R_{z}\sin R_{y}\sin R_{x}-\sin R_{z}\cos R_{x} & \cos R_{z}\sin R_{y}\cos R_{x}+\sin R_{z}\sin R_{x}\\ \sin R_{z}\cos R_{y} & \sin R_{z}\sin R_{y}\sin R_{x}+\cos R_{z}\cos R_{x} & \sin R_{z}\sin R_{y}\cos R_{x}-\cos R_{z}\sin R_{x}\\ -sinR_{y} & \cos R_{y}\sin R_{x} & \cos R_{y}\cos R_{x} \end{array}$$

***X′*** (*x*′, *y*′, *z*′) is the coordinates transformed into the global panoramic coordinate system, and the scale factor *m* defines the distance from the rectified image plane to the projection surface (typically a sphere or cylinder). By combining Equations (5) and (6), we can resolve *m* and ***X′*** for a sphere projection.

In the next step, the collinearity equation based on the multi-camera rig is established. As shown in Figure 4b, the real 3D ray is through *CuP*′, instead of *SuP*, which can be vectorized as $\left(X^{\prime }-T_{r}\right)$. Translating the vector to the global camera coordinate system yields:

(8) $$T_{r}+\lambda \left(X^{\prime }-T_{r}\right)=R^{T}\left(P-T\right)$$

Equation (8) would be the same as the sphere projection (4) when ***T_r_*** is small enough and vanishing. However, for the self-assembly panoramic camera whose ***T_r_*** is too large to ignore, the panoramic camera model is a better choice.

## 3. Line-Based Registration Method

To simplify the transformation in Equation (1), an auxiliary coordinate system is introduced, which is close to the camera-centered coordinate system but still has ***ΔM*** bias. Using ***M*_1_** and ***M*_2_** in Figure 2, LiDAR point ***P_w_*** is transformed in the world coordinate into the auxiliary coordinate ***P***, as is defined in Equation (9), which is further discussed below:

(9) $$P=\left[\begin{array}{cc} R_{2} & T_{2}\\ 0 & 1 \end{array}\right]\left[\begin{array}{cc} R_{1} & T_{1}\\ 0 & 1 \end{array}\right]P_{w}$$

### 3.1. Transformation Model

**Figure 7:**
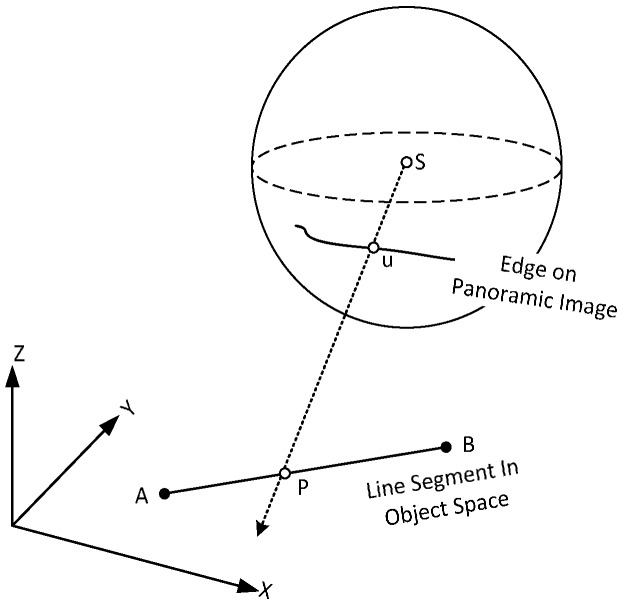
Line-based transformation model on panoramic image.

Suppose a known line segment ***AB*** is given in the world coordinate (actually the auxiliary coordinate in this paper). Its corresponding line in the panoramic image is detected as edge pixels. The projection ray through the perspective panoramic camera center ***C***, an edge pixel *p* on panoramic image, intersects ***AB*** on point ***P***, as illustrated in Figure 7. By letting the line be represented by the two endpoints ***X_A_*** and ***X_B_***, an arbitrary point ***P*** is defined using Equation (10):

(10) $$P=X_{A}+t\left(X_{B}-X_{A}\right)$$

with *t* a scale factor along the line.

Substituting the object point ***P*** in Equation (10) to Equation (4) yields the line-based sphere camera model:

(11) $$\lambda X=R^{-1}\;[(X_{A}-T)+t\left(X_{B}-X_{A}\right)]$$

Further, Equations (2) and (3) are combined and ***P*** is substituted in Equations (10)–(8), yielding the line-based panoramic camera model:

(12) $$T_{r}+\lambda \left(X^{\prime }-T_{r}\right)=R^{-1}\;[(X_{A}-T)+t\left(X_{B}-X_{A}\right)]$$

where ***X*′** can be obtained from Equation (6). The scalar parameter λ and the line parameter *t* are unknown. What we try to resolve is the rotation matrix ***R*** and translation ***T***.

### 3.2. Solution

To get the best alignment between the two datasets, the non-linear least squares method is used to solve the unknowns iteratively. Euclidean distance in the panoramic image coordinate system is used as the similarity metric. Denoting the right-hand term in Equation (11) as ${\left[\bar{X}\;\bar{Y}\;\bar{Z}\right]}^{T}$ and combining Equations (2) and (4), resulting Equation (13):

(13) $$\{\begin{array}{c} u=\left[\tan^{-1}\left(\frac{\bar{X}}{\bar{Y}}\right)\cdot \frac{W}{\pi }+W\right]\cdot \frac{1}{2}\\ v=\left[1-\sin^{-1}\left(\frac{\bar{Z}}{\sqrt{{\bar{X}}^{2}+{\bar{Y}}^{2}+{\bar{Z}}^{2}}}\right)\cdot \frac{2}{\pi }\right]\cdot \frac{H}{2} \end{array}$$

where (*u*, *v*) is the coordinates in the panoramic image coordinate system and (*W*,*H*) is the panoramic image size.

In addition to the six orientation and translation values (X,Y,Z,φ,ω,κ), unknown line parameter *t* must be estimated. Here the right terms are multivariate composite functions ***f****_u_*(***R***, ***T***, *t*) and ***f****_v_*(***R***, ***T***, *t*). Given one pixel on the corresponding lines, the two equations in Equation (11) are formed with one line-parameter *t* introduced. In order to solve the six unknowns, at least six points are needed. If one point per line is used, six pairs of corresponding lines are needed; and if two points per line are used, three pairs of corresponding lines are needed. More than two points on a line does not reduce the rank deficiency but only increases the redundancy.

The equations of *i*-th pair of corresponding lines can be termed by $\{\begin{array}{c} u_{i}=f_{u}\left(R,T,t\right)\\ v_{i}=f_{v}\left(R,T,t\right) \end{array}.$ Defining a parameter vector $X={\left(X,Y,Z,\varphi ,\omega ,\kappa ,t_{1},\cdots ,t_{n}\right)}^{T}$ for *n* pairs of corresponding lines, Equation (13) is then expanded as Equation (14) after linearization by the Taylor series:

(14) $$\{\begin{array}{c} u=u^{0}+\frac{\partial f_{u}}{\partial X}\cdot ∆X+\frac{\partial f_{u}}{\partial Y}\cdot ∆Y+\cdots +\frac{\partial f_{u}}{\partial t_{n}}\cdot ∆t_{n}\\ v=v^{0}+\frac{\partial f_{v}}{\partial X}\cdot ∆X+\frac{\partial f_{u}}{\partial Y}\cdot ∆Y+\cdots +\frac{\partial f_{v}}{\partial t_{n}}\cdot ∆t_{n} \end{array}$$

The above equation is expressed in matrix form as Equation (15):

(15) V=A∆−L

where $∆={\left(∆X_{T},∆Y_{T},∆Z_{T},∆\varphi ,∆\omega ,∆\kappa ,∆t_{1},\cdots ,∆t_{n}\right)}^{T}$ and $L=\left(L_{1};\cdots ;L_{n}\right)$ with $L_{i}=\;{\left(u_{i}-u_{i}^{0},v_{i}-v_{i}^{0}\right)}^{T}$. The coefficient matrix $A\left(A_{1};\cdots ;A_{n}\right)$. are defined as partial derivative of functions $f_{u}$ and $f_{v}$:

(16) $$A_{i}=\left\{\begin{array}{c} \frac{\partial f_{u}}{\partial X},\frac{\partial f_{u}}{\partial Y},\frac{\partial f_{u}}{\partial Z},\frac{\partial f_{u}}{\partial \varphi },\frac{\partial f_{u}}{\partial \omega },\frac{\partial f_{u}}{\partial \kappa },\frac{\partial f_{u}}{\partial t_{1}},\cdots ,\frac{\partial f_{u}}{\partial t_{n}}\\ \frac{\partial f_{v}}{\partial X},\frac{\partial f_{v}}{\partial Y},\frac{\partial f_{v}}{\partial Z},\frac{\partial f_{v}}{\partial \varphi },\frac{\partial f_{v}}{\partial \omega },\frac{\partial f_{v}}{\partial \kappa },\frac{\partial f_{v}}{\partial t_{1}},\cdots ,\frac{\partial f_{v}}{\partial t_{n}} \end{array}\right\}$$

The results can be obtained by solving the normal equation $∆={\left(A^{T}A\right)}^{-1}A^{T}L$. The unknowns X are updated through X←X+∆ iteratively until the elements of ∆ are less than a given threshold. In order to assess the accuracy of the results, the standard deviation is calculated by Equation (17):

(17) $$m_{0}=\pm \sqrt{\frac{V^{T}V}{r}}$$

Here *r* is the number of redundant observations, *r* = (*2* × *n*) − (*n* + *6*). *n* is the number of pixels involved in the transformation, and usually *n = 2m* with *m* pairs of corresponding lines. To handle the mismatch between the LiDAR cloud lines and image lines as well as the occasional large biases in GPS/IMU records, a RANSAC paradigm [29] is applied in iteration to remove the outliers in the corresponding line segments from the LiDAR and the camera.

## 4. Line Feature Extraction from LiDAR

The insufficient density of LiDAR points in a low-cost configuration usually makes 3D line fitting a challenge. The previous works about line extraction tend to use the intersection of two neighboring unparalleled planar patches. However, the methods cannot work well in our case. There are only a few intersections of planar patches in the dataset both due to the point cloud density and flat building facades. Hence we fit linear features directly from cloud points. There are three types of objects containing abundant linear features in the common street-view scenes: buildings, pole-like objects and curbs. In this section, we introduce the methods to fit 3D straight lines from points belonging to the three objects. It is noted that the line-fitting is based on the well classified 3D point cloud achieved by the existing algorithms or software.

### 4.1. Buildings

**Figure 8:**
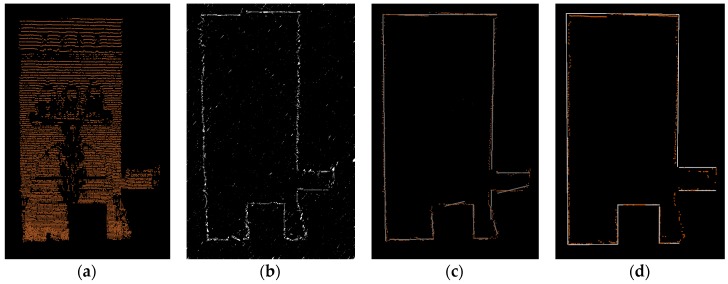
Line segments fitting for building patch: (**a**) projected points; (**b**) boundary points; (**c**) fitting lines using conventional least square method; and (**d**) fitting lines using regularity constraints.

Buildings provide the most reliable straight line features in street view. Given a mobile LiDAR point cloud dataset $P\left\{p_{i}|p_{i}\left(x_{i},y_{i},z_{i}\right)\right\}$ of buildings, a group of 3D line segments $L\left\{l_{j}|l_{j}\left(x_{0j},y_{0j},z_{0j};x_{1j},y_{1j},z_{1j}\right)\right\}$ can be obtained. The detection procedure consists of three steps: (1) apply a region growing segmentation [44] on P and get a set of planar segments $S\left\{s_{k}|s_{k}\left(i_{k1},i_{k2},\cdots ,i_{kn}\right)\right\}$; (2) project the points onto the 3D plane model of segments $s_{k}$ (Figure 8a) and detect the boundary points in each segment $s_{k}$ (Figure 8b); and (3) fit the boundary points into 3D straight border lines with RANSAC (Figure 8c).

In order to overcome the weakness of the traditional least-square regression method [45], certain constraints are introduced: (1) lines must be through the outermost point, instead of the centroid; (2) only the lines close to being vertical or horizontal are considered; and (3) only the lines having sufficient points are considered (Figure 8d). Compared to the line segments detected by [33] (Figure 8c), the fitting lines with constraints are more reliable.

### 4.2. Street Light Poles

**Figure 9:**
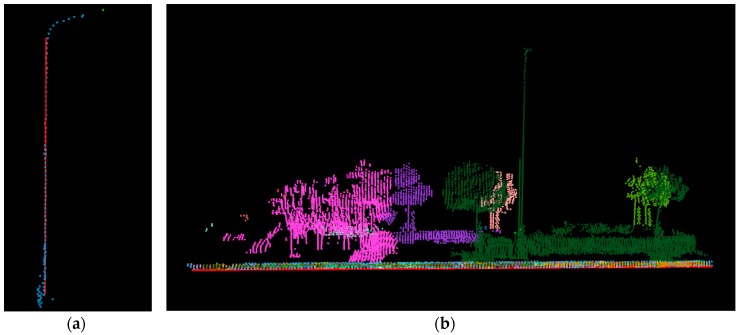
Line segments fitting for (**a**) pole-like objects and (**b**) curbs.

The pole-like segments are labeled and divided into separate objects by spatial connectivity and presented by one or two arrays of points. A percentile-based pole recognition algorithm [46] is adopted to extract the pole objects, which could exclude disrupting structures, such as a flowerbed at the bottom of a light pole and non-pole elements such as lamps. The main steps of the algorithm are as follows: (1) the segment is first sliced into subparts, for which 2D enclosing rectangles and centroids are derived; (2) the deviation of the centroids between neighboring subparts is checked; (3) the diagonal length of the rectangle is checked; (4) the neighboring subparts with the maximum length are kept. The final fitting line segment is defined by the 2D centroids and the minimum and maximum Z values. The fitting results are shown in Figure 9a.

### 4.3. Curbs

Curbs are usually located at a height of 10–20 cm above the road surface and are designed to separate the roads from the sidewalks. The density of the points on the ground is relatively high, and the points on curbs present as narrow stripes which are vertical to the road surface [28]. A curb-line can be approximated as the intersection of the vertical curb with the ground surface. In this paper, the curb-lines in the following steps: (1) the points assigned as curbs are fitted into a plane parallel to the Z-axis under a RANSAC method with direction constraint and the noises are filtered as outliers; (2) the points are fitted into the 2D line segment in the OXY plane, (3) the height of the ground is used as the Z value of the 2D line segment. The fitting results are shown in Figure 9b.

## 5. Experiments and Results

### 5.1. Datasets

**Figure 10:**
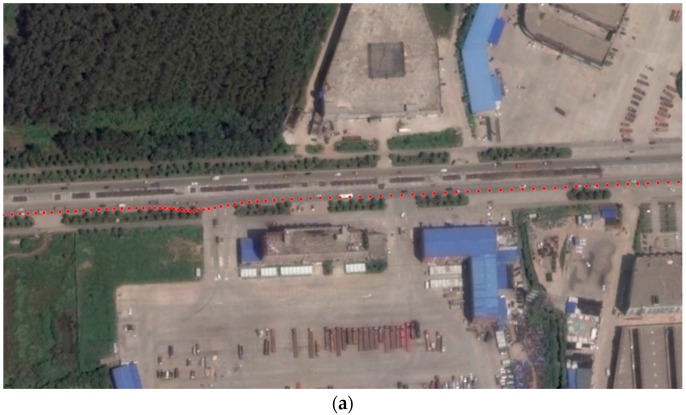

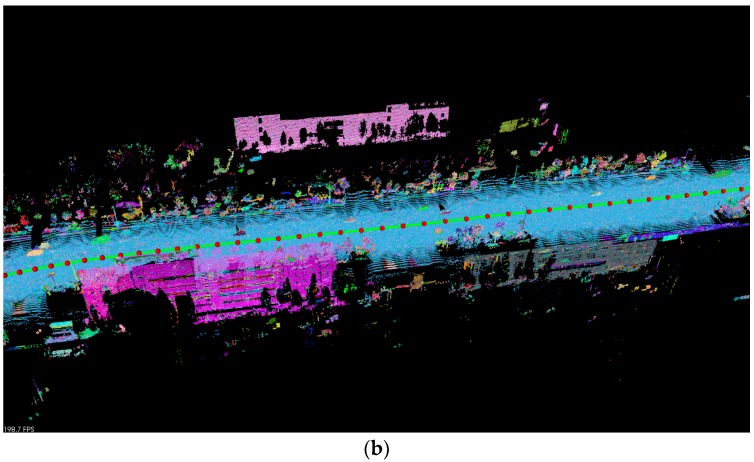
Overview of the test data: (**a**) the test area in Google Earth; (**b**) 3D point cloud of the test area.

The test data were collected on Hankou North Street in the northern part of Wuhan City and included buildings, trees, poles, streets, and moving cars. Figure 10a shows the test area in Google Earth, and Figure 10b shows the 3D point cloud of the test area containing about 1.2 million points, which were previously classified and rendered by classification.

In both subfigures, the red dots are the driving path, and each of the dots is the location where the panoramic camera exposed at an approximate spacing of 7.5 m. The GPS observations were post-processed with RTK [47] technology and can reach an accuracy of up to 0.1 m.

We first extracted a number of three typical line features from the LiDAR dataset. Second, we projected the lines to the rectified mono-images to obtain the corresponding 2D lines, followed by a manual check for eliminating possible one-to-many uncertainty. Then, the proposed registration approach based on the panoramic camera model was applied. A linear feature from LiDAR was defined by two 3D endpoints; and a linear feature from an image was defined as a sequence of pixels in which only two pixels were used in the transformation. Finally, the registration results were assessed based on 2D and 3D visual comparison before and after registration, quantitative evaluation of check points, and statistical evaluation of edge pixels and 3D boundary points.

As mentioned in Section 2.1, the MMS recorded the POS data of the vehicle when it captured an image, while the exterior orientation parameters (EOP) of the camera relative to the vehicle platform coordinate system were acquired in advance through system calibration. The EOP defined the position and rotation of the camera at the instant of exposure with six parameters: three Euclidean coordinates (*X*, *Y*, *Z*) of the projection center and three angles of rotation (φ,ω,κ). Table 1 shows an example of the POS data and the EOP, which correspond to ***M***_1_(***R***_1_, ***T***_1_) and ***M***_2_(***R***_2_, ***T***_2_), respectively, in Equation (9).

Table 2 shows the known parameters of the six cameras in the panoramic camera model. *R_x_*, *R_y_*, *R_z_* are the rotation angles about the X, Y and Z axes, and *T_x_*, *T_y_*, *T_z_* are the translation along the X, Y and Z axes. *x*_0_, *y*_0_ indicate the pixel location of the camera center, and *f* is the focal length. These parameters and definitions of the coordinate systems are discussed in detail in Section 2. These parameters are used in the line-based panoramic camera model (12).

### 5.2. Registration Results

This section analyzes the registration results based on the panoramic camera model in the following steps: registration, visual inspection, and quantitative and statistical evaluation. For comparison purposes, the results from the spherical camera model are presented as well. Table 3 lists the registration results based on the spherical and panoramic camera models. Here the deltas are the corrections after registration, which are the correction term in Equation (15). The root mean square error (RMSE) is the assessment of registration accuracy, which is defined in Equation (17). Both RMSEs were below five pixels. Given an object point 20 m away from the camera center, the error in the object space was about 6 cm.

**Figure 11:**
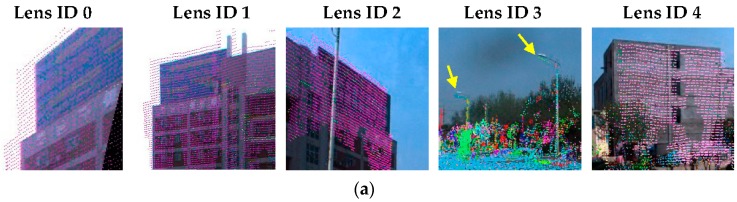

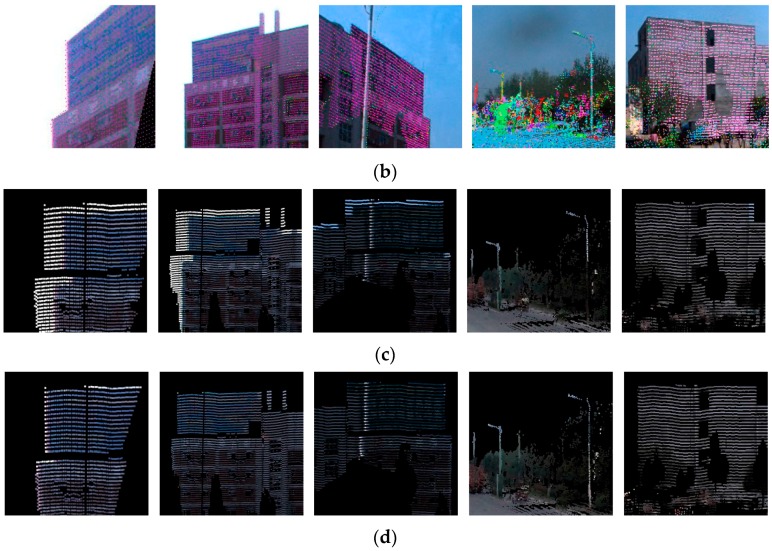
Alignments of two datasets before and after registration based on the panoramic camera model with lens id 0–4. (**a**,**b**) are the LiDAR points projected to a panoramic image before and after registration respectively; (**c**,**d**) are the 3d point cloud rendered by the corresponding panoramic image pixels respectively.

Figure 11a,c are the panoramic images and the labeled LiDAR point cloud before registration, and Figure 11b,d are those after registration with the panoramic camera model, which visually proved that the proposed method effectively removed the displacement between the panoramic image and the LiDAR point cloud (see the borders of buildings in Lens ID 0–2, the two poles marked with yellow pointers in Lens ID 3, and the windows in Lens ID 4).

**Figure 12:**
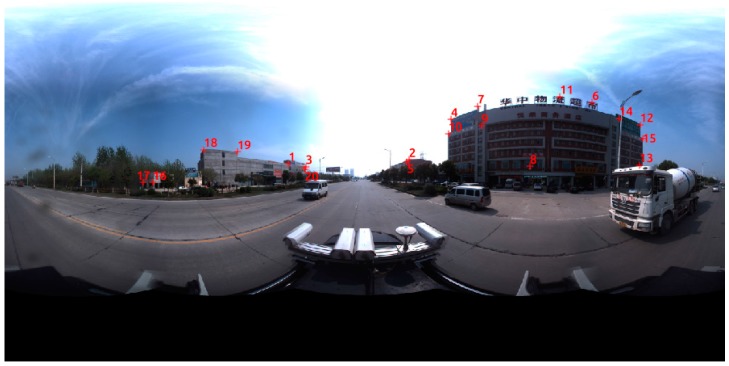
Check points distribution shown on panoramic image. (The meaning of the Chinese characters on the building is Supermarket for Logistics in Central China).

To quantitatively evaluate the registration results based on the panoramic camera model, we manually selected check points both in the LiDAR points and the images according to the following rules: (1) the check points must be correspondent and recognizable in both datasets; (2) the check points should be selected from stationary objects with sufficiently dense LiDAR points; and (3) the check points should be evenly distributed horizontally. As a result, 20 check points were selected (see Figure 12) in the 3D LiDAR point cloud and the panoramic image, whereas 28 corresponding points were selected on the rectified mono-camera images. Please note that there are more check points for images than for LiDAR points because the check points for the panoramic model in the overlapping areas appear twice in adjacent cameras.

**Figure 13:**
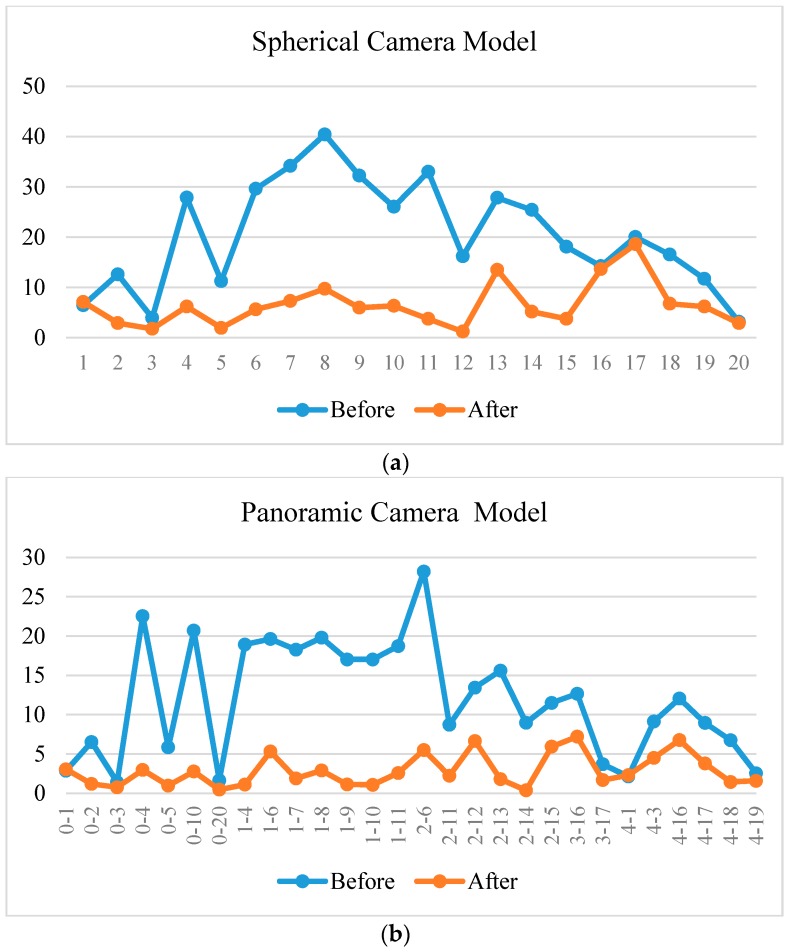
The residuals of the check points before and after registration based on the panoramic camera model. The vertical axis is the residual in pixels; the horizontal axis is (**a**) the ID of the check points and (**b**) the ID of the lens and check points (lens ID—check point ID).

We projected all the check points to images to determine their 2D coordinates before and after registration separately. Then, we calculated the Euclidean distances between the projected 2D points and the image check points as residuals. According to Figure 13, both the spherical and panoramic camera models reduced the residuals significantly, with the latter showing a slight advantage. The average residuals decreased from 12.0 to 2.9 pixels with the panoramic camera model (Figure 13b), and from 20.5 to 6.5 pixels with the spherical camera model (Figure 13a) after registration. The residuals on most check points decreased substantially after registration, and a few of them showed minimal change. The latter occurred for the checks points whose initial residuals were small enough before registration, such as 1, 3, and 20.

**Figure 14:**
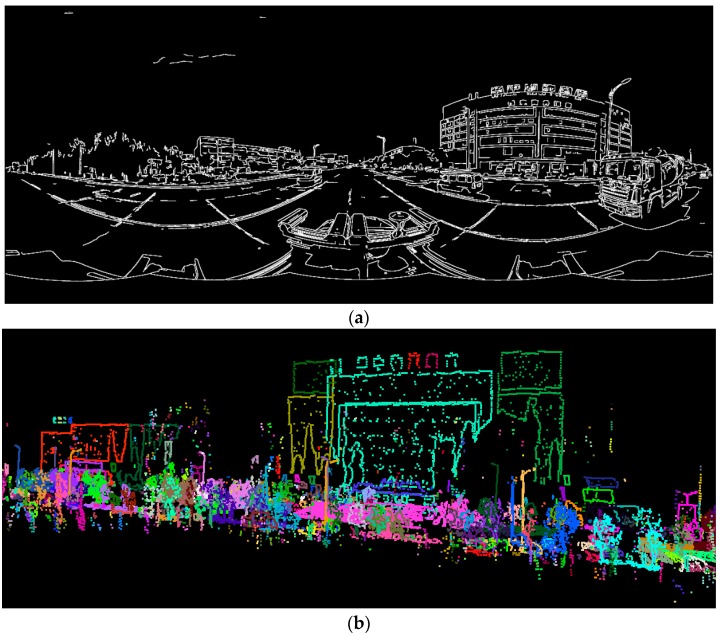
Linear features of the two datasets. (**a**) EDISON edge pixels in the panoramic image; and (**b**) boundary points in LiDAR point cloud.

In order to further evaluate the overall effect of registration, we also calculated the statistical value “*overlap rate*” of the linear features from the two datasets. For the panoramic image, we adopted the EDISON edge detector [48] to extract the edge pixels, as shown in Figure 14a, in which 8 × windows and 20 pixels were used as the min length of the lines to remove noise. For the LiDAR points, we extracted the boundary points using 30 k-nearest neighborhoods, and the results are shown in Figure 14b. From the figures, it can be seen that most of the geometric linear features in the two datasets corresponded. At the same time, some disturbing elements were present, such as the edges due to the color difference in the image and the points from missing data in the LiDAR points.

**Figure 15:**
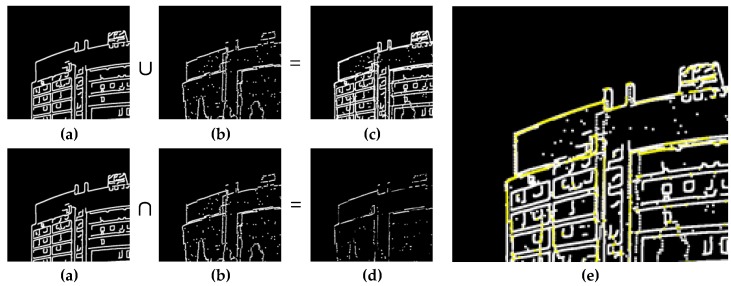
Definition of overlap rate: (**a**) image edge pixels; (**b**) LiDAR projected boundary points; (**c**) union of (a,b); (**d**) overlap of (a,b); (**e**) composition of (c) and highlighted (d).

Figure 15 illustrates the “*overlap rate*” of the linear features from the two datasets. First, we projected the 3D boundary points onto an image and determined the binary image (see Figure 15b). Second, we performed the Overlap and Union operation on the two binary images (see Figure 15a,b) to determine the overlap binary image (see Figure 15c) and the union binary image (see Figure 15d). We then counted the number of non-zero pixels in both binary images: the number in the overlap binary image is *n_o_*, and the number in the union binary image is *n_u_*. Finally, we defined the overlap rate as follows:

(18) $$r=n_{o}/n_{u}$$

If the alignment of the image and the LiDAR point cloud improves, there will be more overlapping linear features, which means $n_{o}$ will increase while $n_{u}$ will decrease and the overlap rate will increase after efficient registration. The results in Table 4 show that all of the overlap rates slightly increased up to 2% after registration. Among the five lenses, Len ID 1 showed the most significant improvement, mainly because part of the image captured by Len ID 1 was a facade containing many linear elements (see Figure 5).

## 6. Conclusions

This paper proposed a line-based registration approach for panoramic images and LiDAR point clouds collected by a MMS. We first established the transformation model between the primitives from the two datasets in the camera-centered coordinate system. Then, we extracted the primitives (three typical linear features) in street view from LiDAR automatically and from panoramic images through a semi-automation process. Using the extracted features, we resolved the relative orientations and translations between the camera and LiDAR.

Compared with other related works, the main contribution of this study is that it focused on the registration between LiDAR and the panoramic camera, which is widely used in a MMS instead of a conventional frame camera. Two types of camera models (spherical and panoramic) were utilized in our registration. The experimental results show that both models were able to remove obvious misalignment between the LiDAR point cloud and the panoramic image. However, the panoramic model achieved better registration accuracy. It is suggested that a suitable camera model may need to be chosen for certain data fusion tasks. For example, for rendering a LiDAR point cloud with acceptable misalignment, the spherical camera model would be adequate while the panoramic camera model may be necessary for high level fusion tasks such as facade modeling.

There are ways to further improve the registration accuracy and automation of the proposed method in future work. First, the errors from the LiDAR point cloud itself could not be overlooked. In our case, the LiDAR points were collected by three laser scanners, whose calibration errors also influenced the registration accuracy. Moreover, finding reliable correspondences between different datasets may need to use local statistical similarity such as mutual information. In addition to geometrical features, utilizing the physical attributes of LiDAR, such as intensity, is also a future research topic.

## Figures and Tables

**Table 1:** POS of the vehicle platform and EOP of the camera aboard.

	POS	EOP
*X* (m)	38,535,802.519	−0.3350
*Y* (m)	3,400,240.762	−0.8870
*Z* (m)	762,11.089	0.4390
φ (°)	0.2483	−1.3489
ω (°)	0.4344	0.6250
κ (°)	87.5076	1.2000

**Table 2:** Parameters of mono-cameras in the panoramic camera model (image size is 1616 × 1232 in pixels).

Lens ID	*R_x_* (Radians)	*R_y_* (Radians)	*R_z_* (Radians)	*T_x_* (m)	*T_y_* (m)	*T_z_* (m)	*x*_0_ (Pixels)	*y*_0_ (Pixels)	*f* (Pixels)
0	2.1625	1.5675	2.1581	0.0416	−0.0020	−0.0002	806.484	639.546	400.038
1	1.0490	1.5620	−0.2572	0.0114	−0.0400	0.0002	794.553	614.885	402.208
2	0.6134	1.5625	−1.9058	−0.0350	−0.0229	0.0006	783.593	630.813	401.557
3	1.7005	1.5633	−2.0733	−0.0328	0.0261	−0.0003	790.296	625.776	400.521
4	−2.2253	1.5625	−0.9974	0.0148	0.0388	−0.0003	806.926	621.216	406.115
5	−0.0028	0.0052	0.0043	0.0010	−0.0006	0.06202	776.909	589.499	394.588

**Table 3:** Registration results based on the spherical and panoramic camera models.

Model	Spherical		Panoramic	
	Deltas	Errors	Deltas	Errors
*X* (m)	−3.4372 × 10^−2^	1.1369 × 10^−3^	3.4328 × 10^−2^	1.0373 × 10^−3^
*Y* (m)	1.0653	1.2142 × 10^−3^	1.0929	1.0579 × 10^−3^
*Z* (m)	1.9511 × 10^−1^	9.9237 × 10^−4^	2.2075 × 10^−1^	8.0585 × 10^−4^
φ (°)	−1.2852 × 10^−2^	1.4211 × 10^−3^	−1.4731 × 10^−2^	1.0920 × 10^−3^
ω (°)	5.8824 × 10^−4^	1.4489 × 10^−4^	1.5866 × 10^−3^	1.2430 × 10^−3^
κ (°)	−7.9019 × 10^−3^	8.4789 × 10^−4^	−6.7691 × 10^−3^	7.7509 × 10^−4^
**RMSE (pixels)**	4.718		4.244	

**Table 4:** Overlap rate based on the panoramic camera model before and after registration.

Lens ID	Before (%)	After (%)
0	7.80	8.29
1	8.31	10.30
2	11.32	11.83
3	9.84	9.90
4	7.42	7.54
